# Morphology-guided deep learning framework for segmentation of pancreas in computed tomography images

**DOI:** 10.1117/1.JMI.9.2.024002

**Published:** 2022-04-04

**Authors:** Touseef Ahmad Qureshi, Cody Lynch, Linda Azab, Yibin Xie, Srinavas Gaddam, Stepehen Jacob Pandol, Debiao Li

**Affiliations:** a Biomedical Imaging Research Institute, Cedars-Sinai Medical Center, Los Angeles, California, United States; b Cedars-Sinai Medical Center, Division of Gastroenterology, Los Angeles, California, United States

**Keywords:** pancreas segmentation, computed tomography pancreas segmentation, morphology priors, deep learning

## Abstract

**Purpose:**

Accurate segmentation of the pancreas using abdominal computed tomography (CT) scans is a prerequisite for a computer-aided diagnosis system to detect pathologies and perform quantitative assessment of pancreatic disorders. Manual outlining of the pancreas is tedious, time-consuming, and prone to subjective errors, and thus clearly not a viable solution for large datasets.

**Approach:**

We introduce a multiphase morphology-guided deep learning framework for efficient three-dimensional segmentation of the pancreas in CT images. The methodology works by localizing the pancreas using a modified visual geometry group-19 architecture, which is a 19-layer convolutional neural network model that helped reduce the region of interest for more efficient computation and removed most of the peripheral structures from consideration during the segmentation process. Subsequently, soft labels for segmentation of the pancreas in the localized region were generated using the U-net model. Finally, the model integrates the morphology prior of the pancreas to update soft labels and perform segmentation. The morphology prior is a single three-dimensional matrix, defined over the general shape and size of the pancreases from multiple CT abdominal images, that helps improve segmentation of the pancreas.

**Results:**

The system was trained and tested on the National Institutes of Health dataset (82 CT scans of the healthy pancreas). In fourfold cross-validation, the system produced an average Dice–SØrensen coefficient of 88.53% and outperformed state-of-the-art techniques.

**Conclusions:**

Localizing the pancreas assists in reducing segmentation errors and eliminating peripheral structures from consideration. Additionally, the morphology-guided model efficiently improves the overall segmentation of the pancreas.

## 1 Introduction

The abdominal computed tomography (CT) scans provide a noninvasive means to assess microlevel morphological features of the pancreas and assists automated diagnosis and treatment plans for several pancreatic disorders. Accurate segmentation of the pancreas in CT images is a prerequisite for reliable quantitative assessment of pancreatic features. Manual outlining of the pancreas can be erroneous, inconsistent, and impractical for large datasets. Automated segmentation is thus highly preferable but is challenged by two major complications: first, the intensity and textural properties of the pancreas and its peripheral organs (e.g., liver, kidney, spleen, etc.) on CT images are highly identical, often resulting in several false positive segmented regions. Second, the morphology of the pancreas is immensely variable and complex, deviating algorithms to specify the exact boundary. This challenge becomes even more severe in the presence of visceral fat tissue around the pancreas that creates fuzzy edges and inconsistent contrast on CT images. All these factors undermine automated segmentation, and therefore, there is a pressing need for a highly robust method that can efficiently overcome these issues currently withholding rigorous assessment of the pancreas.

Literature offers several deep learning (DL)-based techniques that mostly follow deep convolutional neural networks (CNNs) for CT segmentation of the pancreas. Most of these techniques^1^^–^^3^ follow the two-dimensional (2D) approach as there are usually more slices to train the network. Also, the slice thickness of scans can be at times as high as 5 mm (i.e., fewer slices containing pancreas), 2D networks are the most preferred in such scenarios. On the contrary, three-dimensional (3D) networks consider spatial and anatomical information of the pancreas, offering highly stable and robust models.^4^^–^^6^ The 3D approaches, however, require higher computational efficiency and memory compared with 2D models. A hybrid approach,^7^^–^^9^ based on the strategy to integrate output from multiple 2D views to generate 3D segmentation results, has also been adopted, though it still loses some 3D context information important for discriminating the pancreas from its neighboring regions. In addition to DL techniques, common machine learning approaches including random forests^10^ and Gaussian mixture model^11^ have also been used to refine segmentation obtained by DL networks. The mean Dice–SØrensen coefficient (DSC) achieved by existing techniques is as low as 75%, which is far from meeting clinical needs.^12^ However, since the efficiency of the existing models widely varies by the choice of the DL network and the training approach (2D/3D/hybrids) adopted, there is still an opportunity to explore more sophisticated DL networks for improved performance and DSC.

In this work, we developed a multiphase DL 3D framework for accurate segmentation of the pancreas in CT images. The methodology efficiently addresses the aforementioned issues and works by (a) localizing the pancreas using a DL network visual geometry group(VGG)-19,^13^ which eliminates peripheral organs from consideration by determining the general location of the pancreas and down-sizing 3D volume for segmentation of the pancreas, (b) generating soft labels for the pancreas in the localized region using U-net DL architecture,^14^ and (c) updating soft labels using generic prior knowledge on the morphology of the pancreas to help reduce false positive/negative regions and specify the precise boundary of the pancreas in the final segmentation.

The proposed model was trained and tested on an National Institutes of Health (NIH) dataset^15^ (82 CT scans of the healthy pancreas). The fourfold cross-validation was performed where the system produced an average DSC of 88.53% and outperformed existing techniques. The results are highly satisfactory—encouraging researchers to replicate the model and perform validation on their datasets.

The rest of the paper is organized as follows: Sec. 2 describes the proposed methodology, Sec. 3 provides the experimental and implementation details of the methodology, Sec. 4 reports and discusses the results, and Sec. 5 concludes the paper.

## 2 Method

**Fig. 1:**
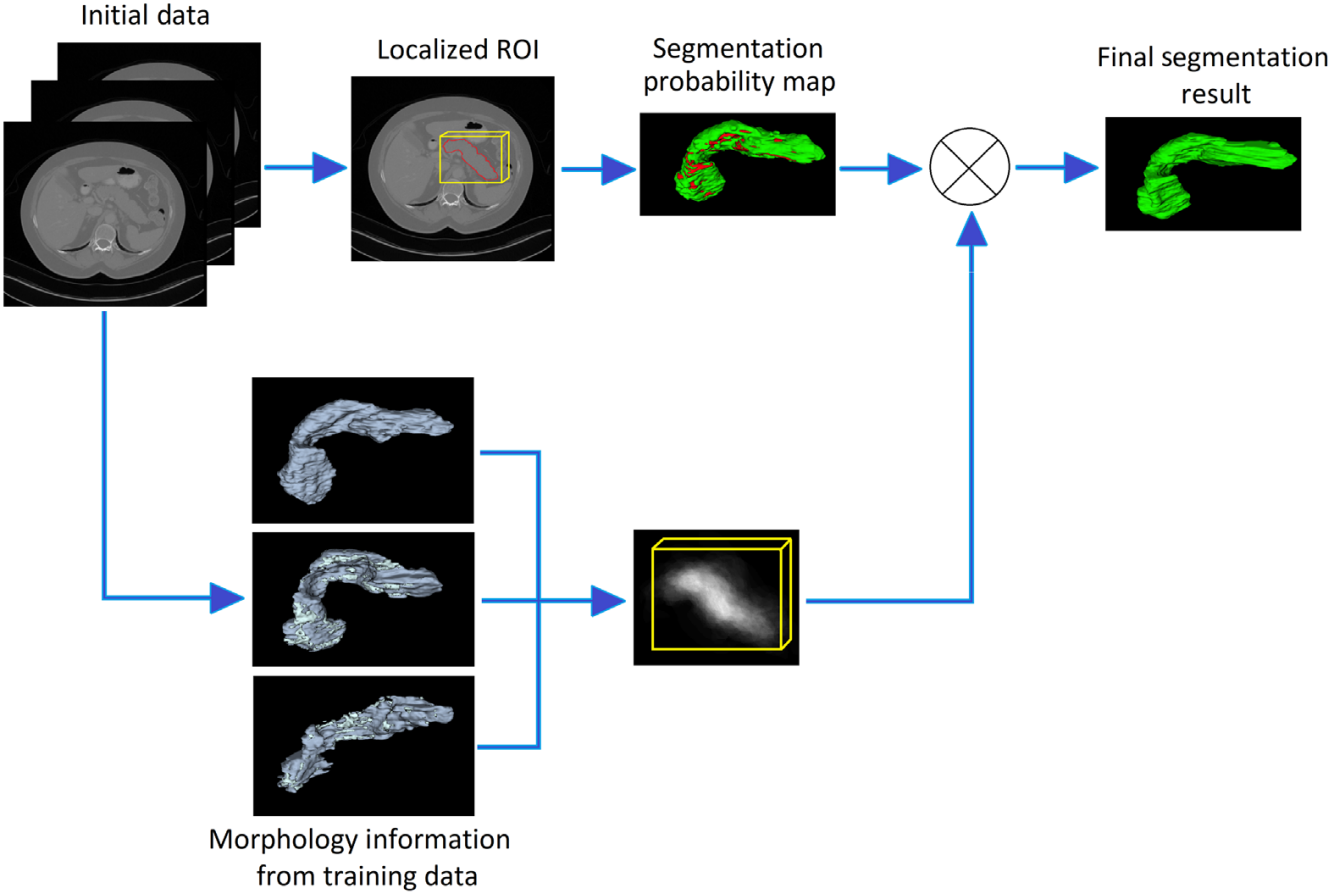
Pictorial description of the proposed methodology.

The proposed methodology consists of three stages, i.e., localizing the pancreas, creating soft labels for the pancreas in the localized region, and upgrading soft labels by integrating the morphology prior to the pancreas to get the final segmentation. Figure 1 is the depiction of the methodology. Methods used in all three stages are explained in the following sections.

### 2.1 Localization of Pancreas

The pancreas is a retroperitoneal organ located in a complex arrangement of abdominal structures. It normally lies on the posterior abdominal wall behind the stomach, across the lumbar (L1 to L2) spine. The pancreas is a long, thin, and relatively small organ in the abdomen. The shape, size, and position of the pancreas are highly affected by several surrounding organs (e.g., liver, stomach, kidneys, and intestines), making it challenging for naïve approaches to correctly outline the pancreas.

Localizing the pancreas in 3D CT scans before performing segmentation has two advantages: first, the peripheral regions presenting identical intensity or textural properties to the pancreas can be easily eliminated as the localizer mainly focuses on the spatial arrangement of all anatomical structures simultaneously in the abdomen rather than detecting the exact boundary of the pancreas. This lessens the chances of getting too many false positive regions during segmentation as the pancreas occupies a very small (e.g., <0.5%) CT volume. Second, specifying the general location of the pancreas allows reducing the dimensionality of the original 3D CT scans, which otherwise requires a high cost of the computational power and memory for training the segmentation models. Lack of hardware resources often leads to limitations in the depth and architecture of the networks, which leads to compromising segmentation accuracy.

**Fig. 2:**
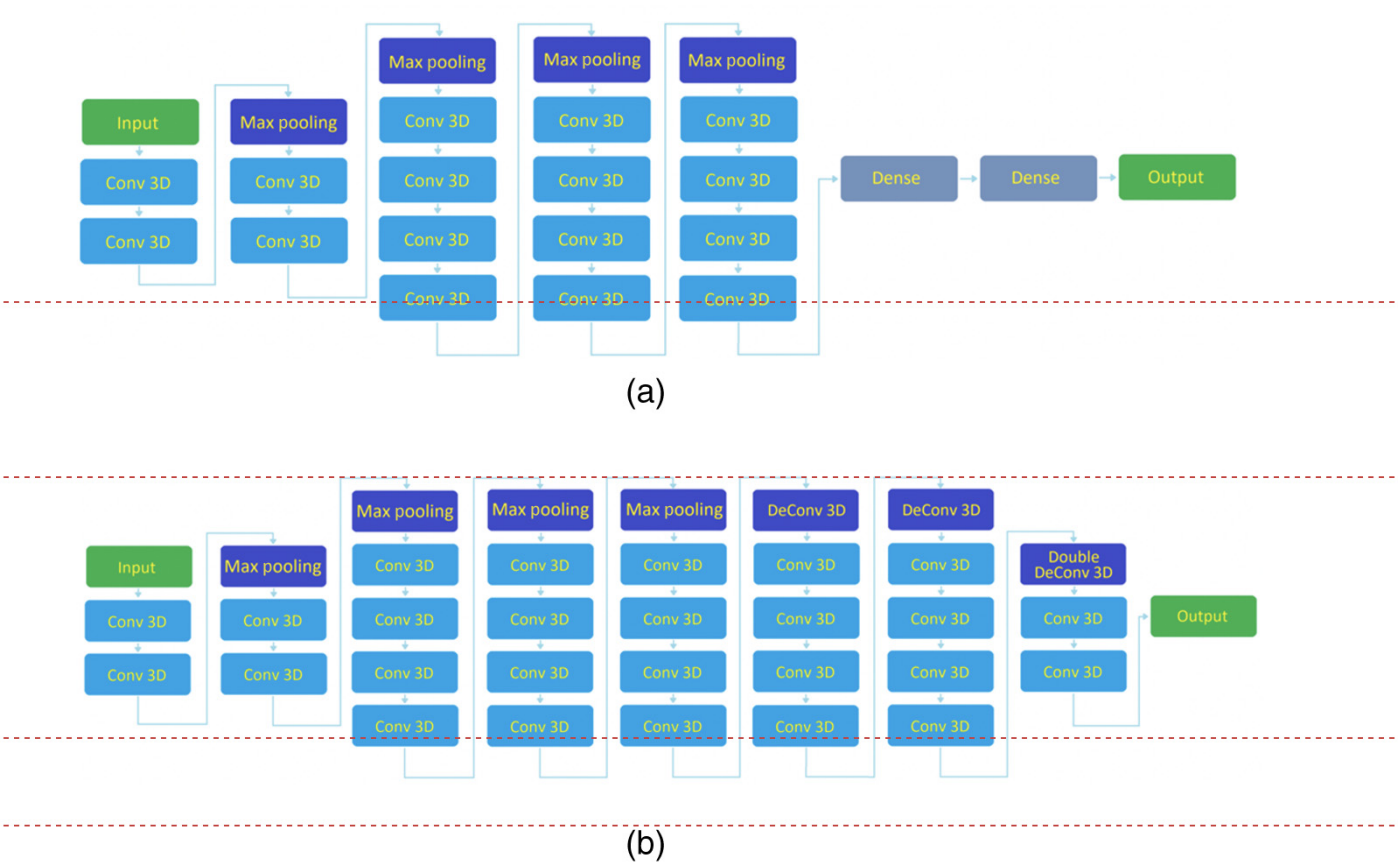
(a) Original VGG-19 network and (b) proposed modified VGG network with restricted layers.

To localize the pancreas in 3D CT scans, we trained the VGG-19 architecture to determine the overall location of the pancreas and down-size the 3D volume of the pancreas for segmentation. The VGG-19 has been frequently utilized in multiple applications for object localization and is among the top five networks with the highest test accuracies for object localization in ImageNet, a benchmark dataset of over 14 million images belonging to 1000 different classes. The original VGG-19 is a 19 layers deep convolutional neural network with 16 convolutional layers, consisting of four maximum pooling layers, six fully connected layers, and a 1000-way SoftMax classifier. However, we deployed a modified version where there are four pooling layers but only three deconvolutional layers, where the third deconvolutional layer has an upsampling factor of four instead of two to maintain image dimensionality (Fig. 2).

**Fig. 3:**
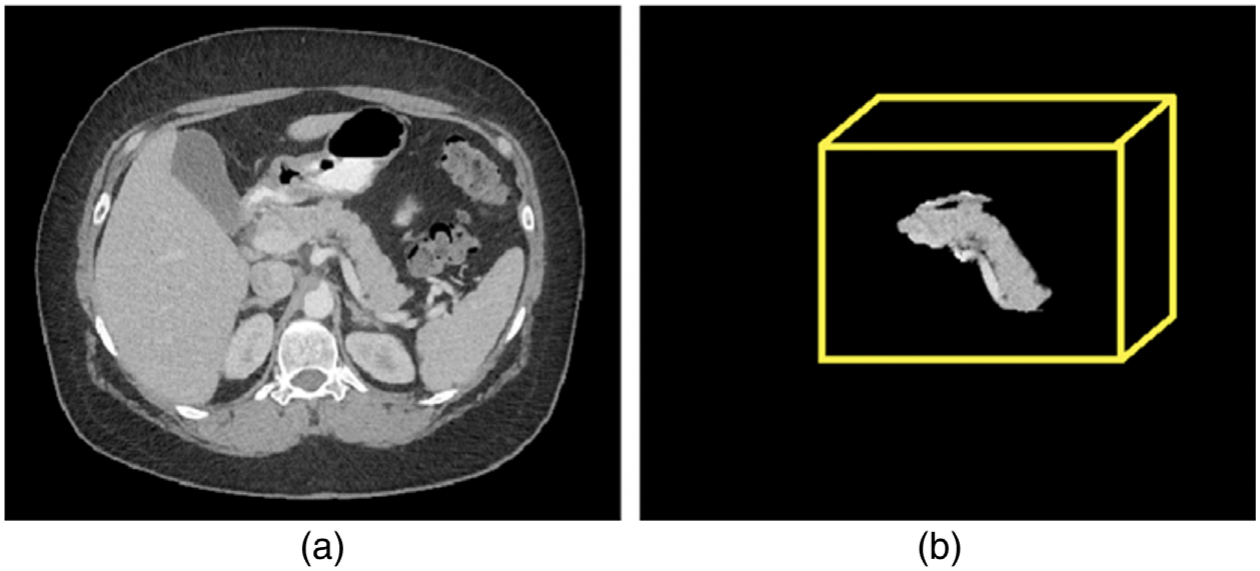
(a) A sample abdominal CT image and (b) the localized 3D pancreas using proposed VGG-19 localizer.

The modified model, although considered a smaller number of features and parameters requiring limited Graphics Processing Unit (GPU) power and memory during training, achieved optimal computational efficiency without compromising on model performance. The model was trained to perform 3D localization of the pancreas by specifying a loose boundary around candidate regions for the pancreas. Subsequently, a normalized bounding box is created to down-sample the volume consisting of candidate regions. Note that the downsampling operation did not alter the original signal intensities or shape of the pancreas. A sample localized pancreas is shown in Fig. 3.

### 2.2 Soft Labels for Pancreas

In the second stage, a standard U-net^14^ architecture was trained to get soft labels for the pancreas in the localized regions obtained in the first stage. The U-net is a DL architecture based on commonly used region-based CNN for fast and precise segmentation of images, particularly when training data are limited or have a great deal of variability. High efficiency and performance of the U-net for segmentation of several small and variable organs in medical images has been observed in previous studies. For each CT abdominal scan, the U-net would generate a soft label, which is a probability map expressing the likelihood of each pixel as the pancreas. Note that it is possible to train the localization and segmentation networks simultaneously as the region of interest (ROI) used here can be generated without the aid of the output in the first stage.

### 2.3 Morphology-Guided Segmentation of Pancreas

In the third stage, a morphology prior, which is a 3D volume template defining the general shape and size of the pancreas, was integrated with the soft labels from the second stage to obtain improved segmentation labels. Having mentioned that the pancreas shares its border with surrounding structures and has a highly variable shape and size, such a template can efficiently assist in improving segmentation accuracy by obtaining a more accurate estimation of the likelihood for each voxel within the ROI to be classified into as background or foreground (pancreas class). We created this morphology prior using gold reference labels (delineation) for pancreases in multiple abdominal CT scans. Labels from several pancreases were combined in a way to yield maximum overlap. The template consists of a single 3D probability map in which the brightness of pixels corresponds to their probability of being associated with the pancreas. For example, pixels lying in the center of morphology prior express high probability (show high intensity) to be part of the pancreas, whereas the pixels usually found farther from the center are less bright (darker) and show less likely to be part of the pancreas. Figure 1 shows a sample template of the morphology prior to the pancreas.

The integration of morphology prior and soft labels was performed by finding the joint probability of the two matrices, updating the original probability map obtained from U-net. The final segmentation was achieved by finding an optimal threshold to perform the classification of pixels into background and foreground based on their estimated probabilities. Our experiment showed that the integration enhanced the overall segmentation accuracy, particularly at the borders of the pancreas.

## 3 Implementation and Experiments

### 3.1 Data for Experiment

Training and validation of the proposed methodology were performed using an NIH pancreas dataset, which contains 82 contrast-enhanced abdominal CT scans. The resolution of each of the CT scans is 512×512  pixels in the x and y axes, respectively, whereas the number of sampling slices on the z axis varies between 181 and 466 with the slice thickness varying between 0.5 and 1.0 mm. The dataset also comes with gold reference labels to outline the pancreases in all scans. In fourfold cross-validation, the dataset was split into four roughly equal size subsets, where three unique subsets were used for model training (∼60 scans) in each fold while the remaining one (∼20) was used for model testing.

### 3.2 Implementing VGG-19 for Pancreas Localization

As a preprocessing step, the images were cropped to remove non-abdomen regions (background), followed by downsampling the volume by a factor of 2 using bilinear interpolation.^16^ The images start off as 512 (x axis) × 512 (y axis) with varying slice numbers that were cropped down to 218×239×288, followed by resizing to fit within the hardware limitations to a size of 96×96×128. The cropping while removing large portions does not remove any of the pancreas pixels in any of the 82 images. The slice range in these images has the most variability and thus the slice dimension was completely left uncropped. This was done to achieve fast processing; for localization we were only required to identify the general location of the pancreas. Also, the intensities in each image were normalized to unity (i.e., 0 to 1) using linear scaling.

The localizer consisted of a modified VGG-19 architecture with four downsampling steps and three upsampling steps where the final upsampling step consists of strides of 4 as opposed to the standard 2; this is done to preserve the dimensionality of the data. This change was made due to hardware limitations. The loss function was set as the mean Dice loss of the pancreas. Network optimization was realized with Adam gradient descent. The learning rate was 1e-6 with a batch size of 1. The maximum epoch number was set to 1500 to keep the best model determined by the performance invalidation data, although the algorithm training generally converged around 500 epochs. The training time on 60 training 3D images took around 8 hours on an NVIDIA GeForce GTX 2080Ti 10 GB GPU.

The output of the localizer was a probability map where any pixel with a probability higher than 0.5 was considered a candidate region for the pancreas. The resultant labels were up-sampled, using bilinear interpolation, to return the label to the original dimensionality and are then used to generate a bounding box. The bounding box was used to specify a localized region (around the candidate regions) in the original image for segmentation to be performed. Padding was applied to the image for a uniform input size for the segmentation process in the following stages.

The labels acquired from the localizer were processed to remove small regions by deleting clusters of pixels that were unconnected from the main body of the label. Removed pixels are input as 0 intensity pixels. Also, the size of the localized images was set to 96×96×128. Padding (empty slices) in the slice dimension has been applied for the cases with the smaller pancreas. The result is a localized image of the pancreas with most non-pancreas regions set to 0 while still maintaining a 100% recall of the pancreas regions across all cases (Fig. 3).

### 3.3 Implementing U-Net to Generate Soft Labels for Pancreas

To generate soft labels for the pancreas, the standard U-net architecture^14^ was deployed. The original model has three downsampling and three upsampling steps. However, due to the large size of our 3D data, we made a modification to increase the depth of the proposed U-net to four downsampling and four upsampling steps. This was done to increase the number of parameters used to segment the pancreas in the larger dataset and increase the overall performance of the model. The U-net takes the 3D volume as input consisting of the localized pancreas with original intensities and most of the non-pancreas regions with 0 intensity.

The loss function was the mean Dice loss of the pancreas. Network optimization was realized with Adam gradient descent whereas the learning rate was 1e-5 with a batch size of 1. The training time, hardware, and number of epochs for U-net were the same as given for the VGG-19 localizer. The outcome of the U-net was a probability map indicating the likelihood of each pixel as pancreas within the bounding box. Both VGG-19 and U-net architectures were implemented in Keras^17^ with the backend of Tensorflow. Table 1 provides the input description of the localization and segmentation model used throughout the process.

**Table 1:** The table provides the matrix sizes and channels for the localization and segmentation model used throughout the process.

Localization				Segmentation			
Input y	Input x	Input z	Channels	Input y	Input x	Input z	Channels
96	96	128	1	144	208	128	1
48	48	64	32	72	104	64	32
24	24	32	64	36	52	32	64
12	12	16	128	18	26	16	128
6	6	8	256	9	13	8	256
12	12	16	128	18	26	16	128

### 3.4 Generating and Applying Morphology Prior

Morphology prior is generated using the gold reference labels of pancreases of training images. The labels were combined in a way to yield maximum overlap between all 60 labels in the training datasets in each fold during fourfold cross-validation.

Let L is the 3D matrix representing the morphology prior, whereas $L_{i}$ (another 3D matrix) denotes the reference label of the pancreas in i’th image in the training set of n images. All pixels belonging to the pancreas in $L_{i}$ represent gray level 1 (i.e., probability 1), whereas all non-pancreatic pixels have 0 gray levels (i.e., probability 0). The pixels with gray level 1 were set with probability 1, whereas nonpancreatic pixels with 0 gray level were set with 0.1 probability (lowest baseline probability) to avoid returning 0 probability during further processing. An overlap was defined as when any two pixels that belong to pancreases from two labels of two different training images have identical spatial (x,y,z) coordinates. The morphology prior L was generated as $L=\overset{n}{\underset{i=1}{\prod }}L_{i}$, such that for any $L_{i-1}$ and $L_{i}$, there is a maximum overlap (intersection) of pancreatic pixels, whereas n is the total number of training images. The pixel probabilities in L are normalized to unity by scaling down between 0 and 1. This process generated a probability map of the general morphology (shape and size) of the pancreas. Padding is applied so the probability map matches the dimensions of the segmentation output.

To integrate morphology prior with the outcome of the U-net model, pixel-wise multiplication of probabilities of morphology prior (L) and the soft labels was performed. This new probability map (resulting matrix) was then scaled to be between 0 and 1 and applied thresholding to obtain optimal segmentation. The optimal threshold to classify pixels into background and foreground based on their estimated probabilities was estimated by-first, excluding all pixels with ≤0.5 probability in the segmentation probability map; this was followed by specifying the optimal threshold that gives the highest mean DSC on the training data in each fold. The average of all optimal thresholds obtained in fourfold was found to be 0.55.

## 4 Results and Discussion

The evaluation of localization of pancreas and segmentation of pancreas (with and without integrating morphology prior) was performed as follows.

The performance of the localizer was assessed by calculating the pixel-wise true positive rate (recall) (TPR) = TP (true positive)/P (positive) and true negative rate (TNR) = TN (true negative)/N (negative) in the localized region specified by the bounding box. The goal of the localization process was to ensure that the box contains as much of pancreatic regions and contains as low a number of nonpancreatic regions as possible. A TPR equals to 1 indicates that the box contains 100% of the pixels that belong to the pancreas. On the other hand, the lower the TNR than 1, the higher number of nonpancreatic regions (false positives) will be in the box. The localizer performed excellently and obtained a mean TPR of value 0.993 in each of fourfold during the validation process, whereas the TNR in the fourfold was observed to be 0.955 on average, as given in Table 2. The gold reference labels were used for calculating the evaluation metrics. It was observed that by localizing the pancreas, the system eliminated 80% to 90% of non-pancreatic regions from the original images, reducing the complexity of the segmentation problem to a significant extent.

**Table 2:** Outcome of the proposed VGG-19 localizer for pancreas in fourfold cross validation.

Fold	Mean TPR (%)	Max TPR (%)	Min TPR (%)	Mean TNR (%)	Min TNR (%)	Max TNR (%)
1	99.22 ± 1.17	100	95.87	97.08 ± 15.92	99.54	91.85
2	99.42 ± 1.17	100	95.86	93.22 ± 13.68	99.44	87.63
3	99.18 ± 1.63	100	93.30	96 ± 16.65	99.57	91.13
4	99.43 ± 1.27	100	94.72	95.62 ± 13.32	99.55	88.34
Mean	99.31	100	95.03	95.48	99.52	89.73

**Fig. 4:**
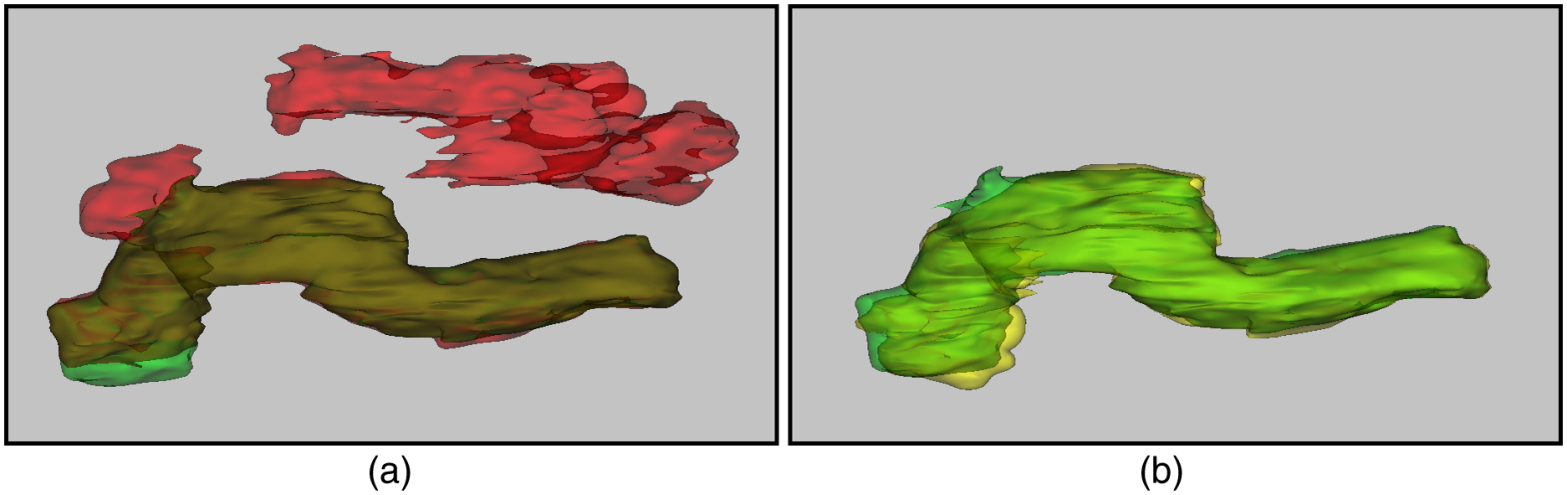
(a) Segmentation obtained before morphology prior application. Red, green, and brown labels indicate false positive, false negative, and true positive regions, respectively. (b) Segmentation of pancreas after applying morphology prior to the pancreas in (a). Green and yellow labels show true positive and false negative regions, respectively.

The segmentation using U-net with and without integrating morphology prior was evaluated by computing mean DSC as segmentation accuracy. The DSC is a similarity metric between the prediction pixels set X and the gold reference label set Y, with the mathematical form of DSC=(2×|X∩Y|)/(|X|+|Y|). Note that the purpose of obtaining DSC for segmentation without integrating morphology prior is only to assess the overall improvement achieved with and without applying morphology prior. The mean DSC achieved by the proposed segmentation model with and without integrating morphology prior in fourfold cross-validation was found 83% and 88.53%, respectively, showing ∼6% improved accuracy when the model incorporates morphology prior. A sample (Fig. 4) shows the outcome after integrating morphology prior to the original soft labels. Also, Table 3 provides insight into how different combinations of the proposed strategies influence the performance of the overall system.

**Table 3:** The ablation table provides the model performance based on different combinations of model components to understand the contribution of each component to the overall segmentation system.

Localization using VGG-19	Morphology prior	Segmentation using U-net	Results (DSC)		
			Min (%)	Max (%)	Mean (%)
Yes	No	No	62.51	99.16	63.61
Yes	Yes	No	73.0	99.21	76.02
No	No	Yes	71.04	97.38	79.20
Yes	No	Yes	77.01	98.3	82.55
No	Yes	Yes	76.08	99.07	84.01
Yes	Yes	Yes	74.62	96.37	88.53

Also, the quantitative results of the proposed segmentation and performance comparison with the existing algorithms for pancreas segmentation published to date are reported in Table 4.

**Table 4:** Comparison of mean DSC achieved by available state-of-the-art methods and the proposed technique for pancreas segmentation using NIH dataset.

Group name	Year	Mean DSC (%)	Min DSC (%)	Max DSc (%)
Cai et al.^3^	2017	82.40	60	90.10
Yu et al.^2^	2018	84.50	62.81	91.02
Oktay et al.^4^	2018	83.10	N/A	N/A
Zhu et al.^18^	2018	84.59	69.62	91.45
Cai et al.^5^	2019	74.30	N/A	N/A
Li et al.^9^	2019	85.70	73.20	91.60
Zhao et al.^17^	2019	85.99	57.20	91.20
Nishio et al.^1^	2020	78.90	N/A	N/A
Xia et al.^19^	2021	79.90	N/A	N/A
Ours		88.53	74.62	96.37

**Fig. 5:**
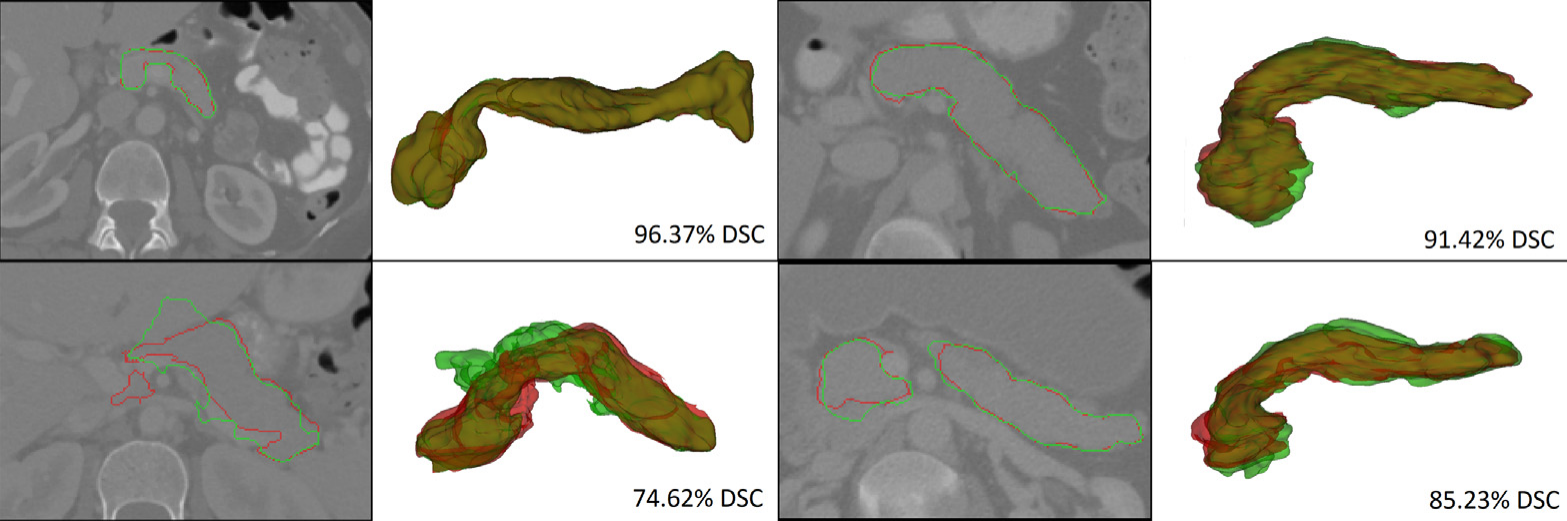
Segmentation results showing the best, average, and worst DSC by the proposed methodology.

Moreover, the proposed method outperformed state-of-the-art algorithms in terms of mean DSC (increased by a factor of ∼3%), implying that the approach is more stable and robust. Furthermore, failure analysis was performed qualitatively. The majority of the incorrectly identified pixels were found on the border of the pancreas, typically where most physicians struggle with delineating between foreground and background. In addition, most of the cases with the lowest DSC had large nonpancreatic regions wrongly classified as pancreas mainly due to their intensities identical to those of the pancreas and their spatial locations, which are in proximity of the pancreas. However, the proposed model efficiently addressed these issues to the best extent in most of the cases and correctly classified these pixels as shown in the sample outcome of the final segmentation in Fig. 5. The overall system remained stable during all experiments, and thus replicable on other datasets.

## 5 Conclusion

A multiphase DL framework for accurate segmentation of the pancreas in CT scans is presented. The method works by localizing the pancreas using the VGG-19 DL network, followed by generating soft labels for the pancreas in the localized region using U-net architecture. The soft labels were then integrated with a 3D volume template defining the general morphology of the pancreas to update soft labels and achieve improved segmentation. The model was trained and tested on an NIH dataset of 82 contrast-enhanced CT scans and produced highly satisfactory results, reaching a mean DSC of 88.53% in fourfold cross-validation and outperforming the state-of-the-art techniques.
